# Supplementary material for: Microencapsulated Limosilactobacillus reuteri Encoding Lactoferricin-Lactoferrampin Targeted Intestine against Salmonella typhimurium Infection
Source: Nutrients. 2023 Dec 18;15(24):5141. doi: 10.3390/nu15245141 (PMC10745908; doi:10.3390/nu15245141)
Supplement: Supplementary file 1 [file nutrients-15-05141-s001.zip › revised Supplementary-nutrients-2745405/Supplementary.docx]

Article

Microencapsulated *Limosilactobacillus reuteri* Encoding
Lactoferricin-Lactoferrampin Targeted Intestine against
*Salmonella typhimurium* Infection

| **Citation:** Wang, X.; Xie, W.; Cai, L.; Han, C.; Kuang, H.; Shao, Y.;  Zhang, S.; Zhang, Q.; Li, J.; Cui, W.; et al. Microencapsulated  *Limosilactobacillus reuteri* Encoding Lactoferricin-Lactoferrampin  Targeted Intestine against *Salmonella typhimurium* Infection. *Nutrients* **2023**, *15*, x. https://doi.org/10.3390/ xxxxx  Academic Editor: Toshifumi Ohkusa  Received: 14 November 2023  Revised: 7 December 2023  Accepted: 15 December 2023  Published: date  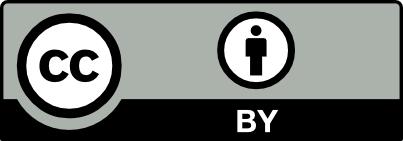  **Copyright:** © 2023 by the authors. Submitted for possible open access publication under the terms and conditions of the Creative Commons Attribution (CC BY) license (https://creativecommons.org/licenses/by/4.0/). |
| --- |

Xueying Wang ^1,†^, Weichun Xie ^1,†^, Limeng Cai ^1^, Chuang Han ^1^, Hongdi Kuang ^1^, Yilan Shao ^1^, Senhao Zhang ^1^,
Qi Zhang ^1^, Jiaxuan Li ^1^, Wen Cui ^1^, Yanping Jiang ^1,^* and Lijie Tang ^1,2,^*

^1^ College of Veterinary Medicine, Northeast Agricultural University, Harbin 150030, China;
tycoon28644@163.com (X.W.); xieweichun_neau@163.com (W.X.); clmyoyo@163.com (L.C.);
hanchuang3198@163.com (C.H.); 18246360750@163.com (H.K.); shaoyilan@neau.edu.cn (Y.S.); zhang_sen_hao@163.com (S.Z.); a502694221@foxmail.com (Q.Z.); lijiaxuan.1993@163.com (J.L.);
cuiwen@neau.edu.cn (W.C.)

^2^ Heilongjiang Key Laboratory for Animal Disease Control and Pharmaceutical Development, Northeast Agricultural University, Harbin 150030, China

***** Correspondence: jiangyanping@neau.edu.cn (Y.J.); tanglijie@163.com or tanglijie@neau.edu.cn (L.T.);
Tel.: +86-451-5519-0385 (Y.J.); +86-451-5519-0824 (L.T.)

^†^ These authors contributed equally to this work.

**Abstract:** *Salmonella* *enterica* serovar Typhimurium (*S. typhimurium*) is an important foodborne pathogen that infects both humans and animals and develops acute gastroenteritis. As porcine intestines are relatively similar to the human ones due to their relatively similar sizes and structural similarity, *S. typhimurium* causes analogous symptoms in both. Novel strategies for controlling *S. typhimurium* infection are also desired, such as mucosal-targeted delivery of probiotics and antimicrobial peptides. The bovine lactoferricin-lactoferrampin-encoding *Limosilactobacillus reuteri* (LR-LFCA) strain improves intestinal barrier function by strengthening the intestinal barrier. Weaned piglets were selected for oral administration of microencapsulated LR-LFCA (microcapsules entrap LR-LFCA into gastro-resistant polymers) and then infected with *S. typhimurium* for 3 days. We found that orally administering microencapsulated LR-LFCA to weaned piglets attenuated *S. typhimurium*-induced production of inflammatory factors in the intestinal mucosa by inhibiting the nuclear factor-kappa B (NF-κB) and P38 mitogen-activated protein kinases (MAPK) signaling pathway. Moreover, microencapsulated LR-LFCA administration significantly suppressed the oxidative stress that may correlate with gut microbiota (reduced *Salmonella* population and increased α-diversity and *Lactobacillus* abundance) and intestinal function (membrane transport and metabolism). Our work demonstrated that microencapsulated LR-LFCA effectively targeted intestine delivery of *Lactobacillus* and antimicrobial peptides and modulated gut microbiota and mucosal immunity. This study reveals a novel targeting mucosal strategy against *S. typhimurium* infection.

**Keywords:** LFCA; *Salmonella typhimurium*; *Limosilactobacillus reuteri*; microcapsules;
intestinal barrier

**Table S1.** The feed formula for piglets.

| **Composition (%)** | **Normal Diet** |
| --- | --- |
| Vit-Min Premix | 0.72 |
| Choline chloride | 0.12 |
| L-lysine | 0.25 |
| DL-methionine | 0.06 |
| Whey powder | 1.50 |
| D-glucose | 1.50 |
| Sodium chloride | 0.35 |
| Monocalcium phosphate | 1.30 |
| Calcium carbonate | 1.20 |
| Corn | 55.00 |
| Soybean meal | 33.00 |
| Fish meal | 5.00 |

**Table S2.** Primers for PCR and Real-time quantitative PCR.

| **Gene** | **Primer Sequences** |
| --- | --- |
| 16S rRNA gene's V3 and V4 regions | F: 5′-CCTACGGGNGGCWGCAG-3′  R: 5′-GGACTACHVGGGTATCTAAT-3′ |
| MyD88 | F: 5’-CTGCCGTCGGATGGTAGT-3’  R: 5’-CAGTGATGAACCGCAGGAT-3’ |
| TRIF | F: 5’-CAAGGCCAAGTGGAGGAAGGAAC-3’  R: 5’-AACTGCGTCTGGTAGGACAGGTAG-3’ |
| TRAF6 | F: 5’-CCAGAGACCCACAATCCCAC-3'  R: 5’-TGGAGACCTCACAGCGTACT-3’ |
| p38 MAPK | F: 5’-AAGACTCGTTGGAACCCCAG-3'  R: 5’-TCCAGCAAGTCAACAGCCAA-3’ |
| AP-1 | F: 5’-AGGCGGAGAGGAAGCGTATGAG-3'  R: 5’-CTGAGCATGTTGGCGGTGGAC-3’ |
| C-Fos | F: 5’-CTGAGATCGCCAACCTGCTGAAG-3’  R: 5’-CAGATCAAGGGAAGCCACAGACATC-3’ |
| c-JUN | F: 5’-ATGACCCTGAACCTGGCTGACC-3’  R: 5’-GTGCCCGTTACTGGACTGGATTATC-3’ |
| IKK-α | F: 5’-AGAGTTCTGCTCGGTCCCTTGTAG-3’  R: 5’-CTTGAGGAGTTACCACGCATGACAG-3’ |
| IKKγ | F: 5’-GACGGAACAAGCACTTAGGGACAG-3’  R: 5’-CTTTGGTGGCGACAGATGACAGAG-3’ |
| NF-κB p50 | F: 5’-GCACCACCTATGATGGAACTACACC-3’  R: 5’-ACGAGTCATCCAGGTCATACAGAGG-3’ |
| GAPDH | F: 5’-CCACTTCCGGGGCACTGTCA-3’  R: 5’-AGCACCAGCATCTGCCCACT-3’ |

**Table S3.** Antibiotic activity of LR-LFCA against pathogenic bacteria.

| **The tested strains** | **The size of the bacteriostatic circle diameter (cm)** | |
| --- | --- | --- |
|  | LR-LFCA | LR-CON |
| *S. aureus* CVCC546 | 1.17±0.09 | 0.69±0.05 |
| *E. coli* CVCC10141 | 0.91±0.08 | 0.66±0.06 |
| *S. typhimurium* SL1344 | 1.09±0.11 | 0.68±0.05 |

**Table S4.** Effects of administering LR-LFCA microcapsules on the performance and diarrhea prevalence in weaned piglets.

| **Items** | **LR-LFCA** | **LR-CON** | **CON** |
| --- | --- | --- | --- |
| Average daily gain (ADG/g) | 486.60±6.25^a^ | 388.62±7.78^b^ | 272.68±2.32^c^ |
| Average daily feed intake (ADFI/g) | 603.38±7.75^a^ | 519.28±10.19^b^ | 368.12±3.13^c^ |
| The feed to gain ratio (F/G) | 1.24±0.06^a^ | 1.31±0.10^a^ | 1.35±0.05^a^ |
| Diarrhea rate (%) | 11.9 | 14.2 | 18.5 |

Note: Data are represented as the mean ± SD. Different lowercase letters represent P < 0.05.

**Table S5.** Effect of the oral administration of microencapsulated LR-LFCA on physiological indices.

| **Items** | **LR-LFCA** | **LR-CON** | **CON** | **Reference range** |
| --- | --- | --- | --- | --- |
| WBC (10^9^/L) | 7.91±0.48^a^ | 7.95±0.04^a^ | 6.21±0.01^a^ | 5.00~8.00 |
| Neu (10^9^/L) | 5.46±0.15^a^ | 4.83±0.12^a^ | 5.00±0.34^a^ | 4.48~7.52 |
| Lymph (10^9^/L) | 11.01±1.20^a^ | 11.25±1.11^a^ | 14.00±1.80^a^ | 6.60~18.70 |
| Neu (%) | 26.99±0.81^b^ | 21.83±0.43^c^ | 47.71±6.81^a^ |  |
| Lymph (%) | 61.03±0.57^a^ | 62.64±0.56^a^ | 45.51±2.39^b^ |  |
| MONO (10^9^/L) | 1.23±0.18^a^ | 1.08±0.04^a^ | 1.08±0.14^a^ | 0.3~1.25 |
| EOS (10^9^/L) | 0.6±0.01^a^ | 0.36±0.01^a^ | 0.21±0.013^a^ | 0.20~1.10 |
| RBC (10^12^/L) | 6.84±0.06^a^ | 7.93±0.01^a^ | 6.25±0.03^a^ | 5.00~8.00 |
| HCT (%) | 49.35±0.05^a^ | 50.07±0.87^a^ | 37.08±0.06^b^ | 32.0~50.0 |
| HGB (g/L) | 14.46±0.03^a^ | 14.36±0.13^a^ | 11.54±0.26^a^ | 10.7~16.7 |
| MCV (fL) | 61.22±0.02^a^ | 62.75±1.45^a^ | 59.38±5.41^b^ | 50.0~68.0 |
| MCH (pg) | 17.45±0.15^a^ | 18.00±0.10^a^ | 18.42±0.57^a^ | 17.0~21.0 |

Note: Different lowercase letters represent P < 0.05.

**Table S6.** Effect of the oral administration of microencapsulated LR-LFCA on serum biochemical parameters.

| **Items** | **LR-LFCA** | **LR-CON** | **CON** | **Reference range** |
| --- | --- | --- | --- | --- |
| GLU | 7.02±0.6^a^ | 7.255±0.365^a^ | 6.78±0.45^a^ | 3.89~7.94 |
| CREA | 87.5±11.5^b^ | 117±17^a^ | 93±12^b^ | 44~159 |
| BUN | 7.55±0.65^a^ | 5.8±0.9^a^ | 9.7±0.8^a^ | 2.5~9.6 |
| BUN/CREA | 22±5.00^a^ | 13±4^b^ | 26±3^a^ |  |
| TP | 60.5±0.50^a^ | 58±2^a^ | 61±1.5^a^ | 52~82 |
| ALB | 34.00±1.00^a^ | 36±1^a^ | 34±1^a^ | 22~39 |
| GLOB | 26.5±0.5^a^ | 22±1^a^ | 27±1^a^ | 22~45 |
| ALB/GLOB | 1.25±0.05^a^ | 1.65±0.05^a^ | 1.2±0.05^a^ |  |
| ALT | 56±3^a^ | 42±5^b^ | 54±4^a^ | 10~100 |
| AST | 43±22^a^ | 46±16^a^ | 49±7^a^ | 0~50 |
| CHOL | 2.855±0.315^a^ | 2.935±0.135^a^ | 3.19±0.2^a^ | 2.84~8.27 |
| TRIG | 0.57±0.15^a^ | 0.38±0.05^a^ | 0.97±0.04^a^ | 0.11~1.13 |

Note: Different lowercase letters represent P < 0.05.

**Table S7.** Effect of the oral administration of microencapsulated LR-LFCA on the physiological indices of weaned piglets after *S. typhimurium* challenge.

| **Items** | **ST+LR-LFCA** | **ST+LR-CON** | **ST** | **CON** | **Reference range** |
| --- | --- | --- | --- | --- | --- |
| WBC (10^9^/L) | 6.99±1.93^b^ | 11.10±3.28^b^ | 24.94±1.88^a^ | 7.09±0.03^b^ | 5.00~8.00 |
| Neu (10^9^/L) | 6.43±1.88^a^ | 8.14±0.20^a^ | 11.02±1.19^a^ | 5.66±1.08^a^ | 4.48~7.52 |
| Lymph (10^9^/L) | 11.02±2.7^a^ | 12.2±0.02^a^ | 7.53±2.29^b^ | 15.06±4.92^a^ | 6.60~18.70 |
| Neu (%) | 26.29±9.60^b^ | 31.12±0.02^b^ | 54.52±5.08^a^ | 21.2±3.2^c^ |  |
| Lymph (%) | 53.88±3.28^b^ | 45.18±0.32^c^ | 37.26±2.23^d^ | 62.4±1.20^a^ |  |
| MONO (10^9^/L) | 3.50±1.30^a^ | 6.28±0.09^a^ | 1.54±0.73^a^ | 1.42±0.47^a^ | 0.3~1.25 |
| EOS (10^9^/L) | 0.14±0.10^a^ | 0.11±0.01^a^ | 0.34±0.25^a^ | 0.17±0.10^a^ | 0.20~1.10 |
| RBC (10^12^/L) | 7.62±0.21^a^ | 7.30±0.10^a^ | 5.52±1.71^a^ | 7.15±0.67^a^ | 5.00~8.00 |
| HCT (%) | 13.48±0.21^a^ | 13.00±0.50^a^ | 10.29±3.50^a^ | 12.86±0.13^a^ | 10.7~16.7 |
| HGB (g/L) | 46.05±0.05^a^ | 48.43±0.43^a^ | 41.45±17.25^a^ | 43.00±0.10^a^ | 32.0~50.0 |
| MCV (fL) | 60.31±1.51^b^ | 65.69±0.11^a^ | 71.41±0.16^a^ | 57.16±2.16^b^ | 50.0~68.0 |
| MCH (pg) | 17.7±0.20^a^ | 17.7±0.7^a^ | 18.17±0.04^a^ | 17.03±0.66^a^ | 17.0~21.0 |

Note: Different lowercase letters represent P < 0.05.

**Table S8.** Effect of the oral administration of microencapsulated LR-LFCA on serum biochemical parameters of weaned piglets after *S. typhimurium* challenge.

| **Items** | **ST+LR-LFCA** | **ST+LR-CON** | **ST** | **CON** | **Reference range** |
| --- | --- | --- | --- | --- | --- |
| GLU | 5.56±0.49^a^ | 4.56±0.35^a^ | 10.555±0.775^a^ | 6.31±0.50^a^ | 3.89~7.94 |
| CREA | 97.5±17.50^b^ | 124±10^a^ | 92±7^b^ | 98.0±8^b^ | 44~159 |
| BUN | 6.7±0.20^a^ | 9.10±0.07^a^ | 3.7±0.30^a^ | 8.3±0.50^a^ | 2.5~9.6 |
| BUN/CREA | 17.5±3.50^a^ | 18±2.50^a^ | 10±2^a^ | 21.0±2^a^ |  |
| TP | 71.5±1.50^a^ | 68±1.50^a^ | 64.5±1.50^a^ | 63.0±1^a^ | 52~82 |
| ALB | 36±1^a^ | 32±2.50^a^ | 32.5±1.50^a^ | 37.0±1.50^a^ | 22~39 |
| GLOB | 38±3^a^ | 35±2^a^ | 32±2^a^ | 26.0±1.50^b^ | 22~45 |
| ALB/GLOB | 1.25±0.15^a^ | 0.9±0.10^a^ | 1.05±0.05^a^ | 1.4±0.15^a^ |  |
| ALT | 45.5±3.50^b^ | 45±2.80^b^ | 37.5±3.50^b^ | 85±2.50^a^ | 10~100 |
| AST | 86±11^c^ | 151.5±15^b^ | 186±12.50^a^ | 40±13^d^ | 0~50 |
| CHOL | 2.02±0.29^a^ | 1.69±0.18^a^ | 1.185±0.45^a^ | 2.91±0.27^a^ | 2.84~8.27 |
| TRIG | 1.105±0.24^a^ | 1.12±0.15^a^ | 0.695±0.12^a^ | 0.99±0.15^a^ | 0.11~1.13 |

Note: Different lowercase letters represent P < 0.05.

**Table S9.** Data for linear discriminant analysis (LDA) in the CON and ST groups.

**Table S10.** Data for linear discriminant analysis (LDA) in the ST and ST+LR-LFCA groups.


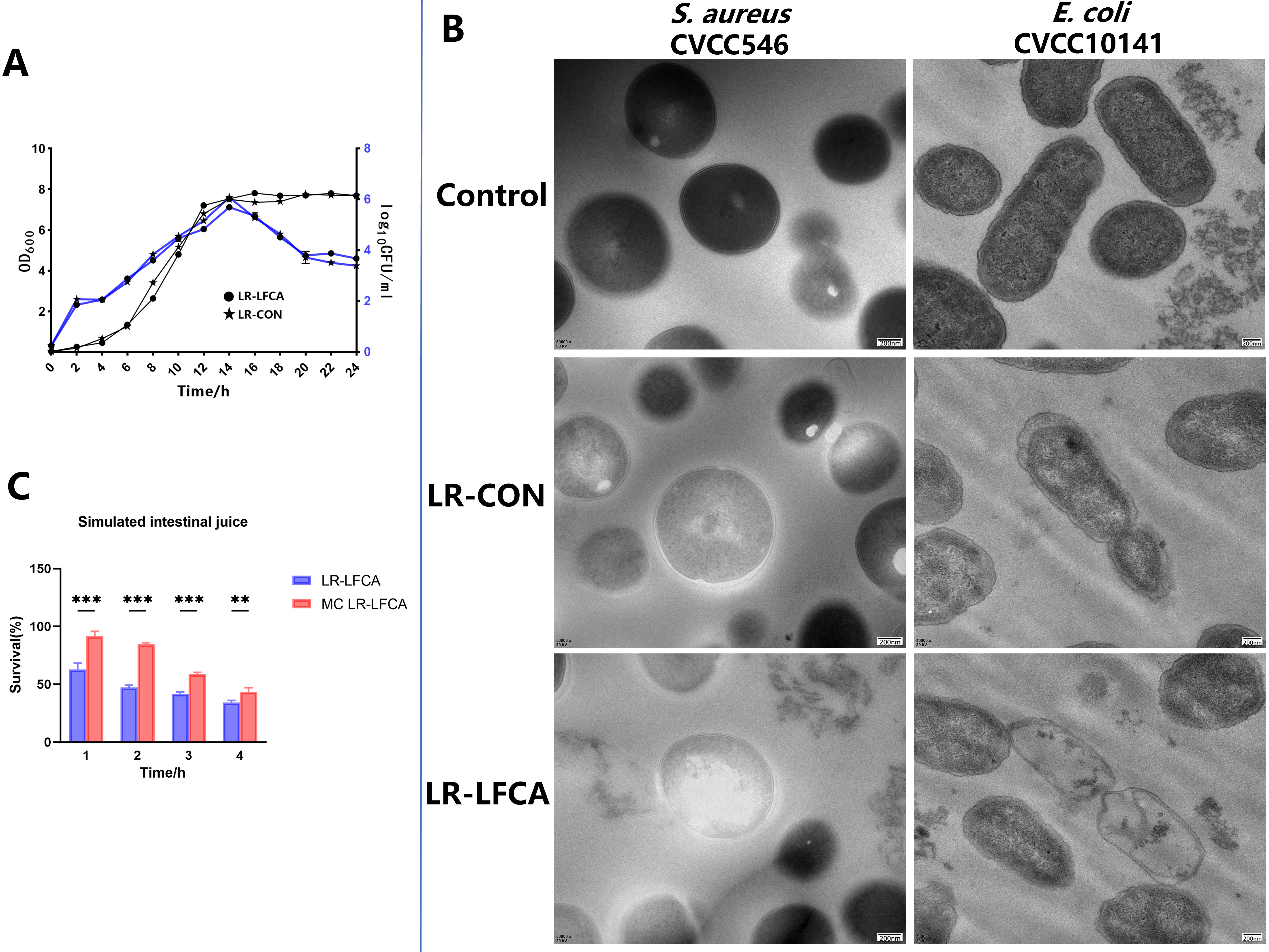


**Figure S1.** Growth curves, antimicrobial activity of LR-LFCA and stress resistance of microencapsulated LR-LFCA. (A) Bacterial growth curve of LR-LFCA and LR-CON. OD values were determined at 600 nm using a microplate reader. (B) Ultrastructural damage in bacteria treated with cell lysates (5 µg protein) from LR-LFCA and LR-CON. Control, bacteria treated with PBS; LR-CON, bacteria treated with cell lysates from LR-CON; LR-LFCA, bacteria treated with cell lysates from LR-LFCA. Cells were analyzed by electron microscopy. (C) Survival of Microencapsulated LR-LFCA in simulated intestinal juice. **P< 0.01; ***P< 0.001.


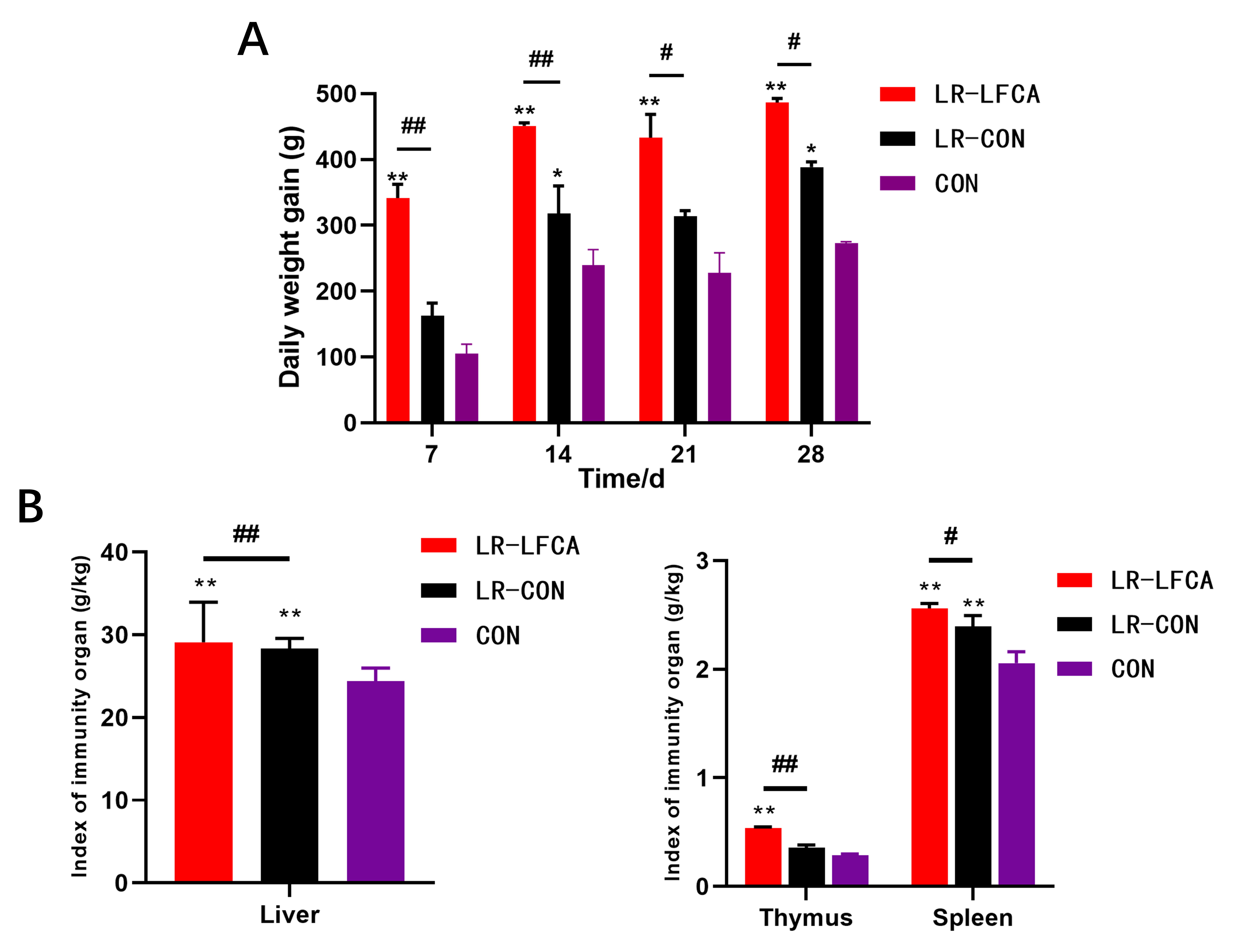


**Figure S2.** Effect of the oral administration of microencapsulated LR-LFCA on the average daily body weight gain and organ index of piglets. (A) Effect of the oral administration of microencapsulated LR-LFCA on the average daily body weight gain of piglets. The bodyweight of piglets was recorded daily for 28 days, calculating the average daily gain (ADG) on days 7, 14, 21, 28 following the oral administration of microencapsulated LR-LFCA. (B) Weaned piglets received continuous oral LR-LFCA administration for a mean of 21 days, and then, we stopped treatment for up to 7 days. Subsequently, we examined the weight of the spleen, thymus, and liver. The relative weights of the organs (spleen, thymus, and liver) were calculated using the following formula: relative organ weight = organ weight (g) / terminal body weight (kg). *P< 0.05 vs CON; **P< 0.01 vs CON; #P< 0.05 vs LR-CON; ##P<0.01 vs LR-CON.


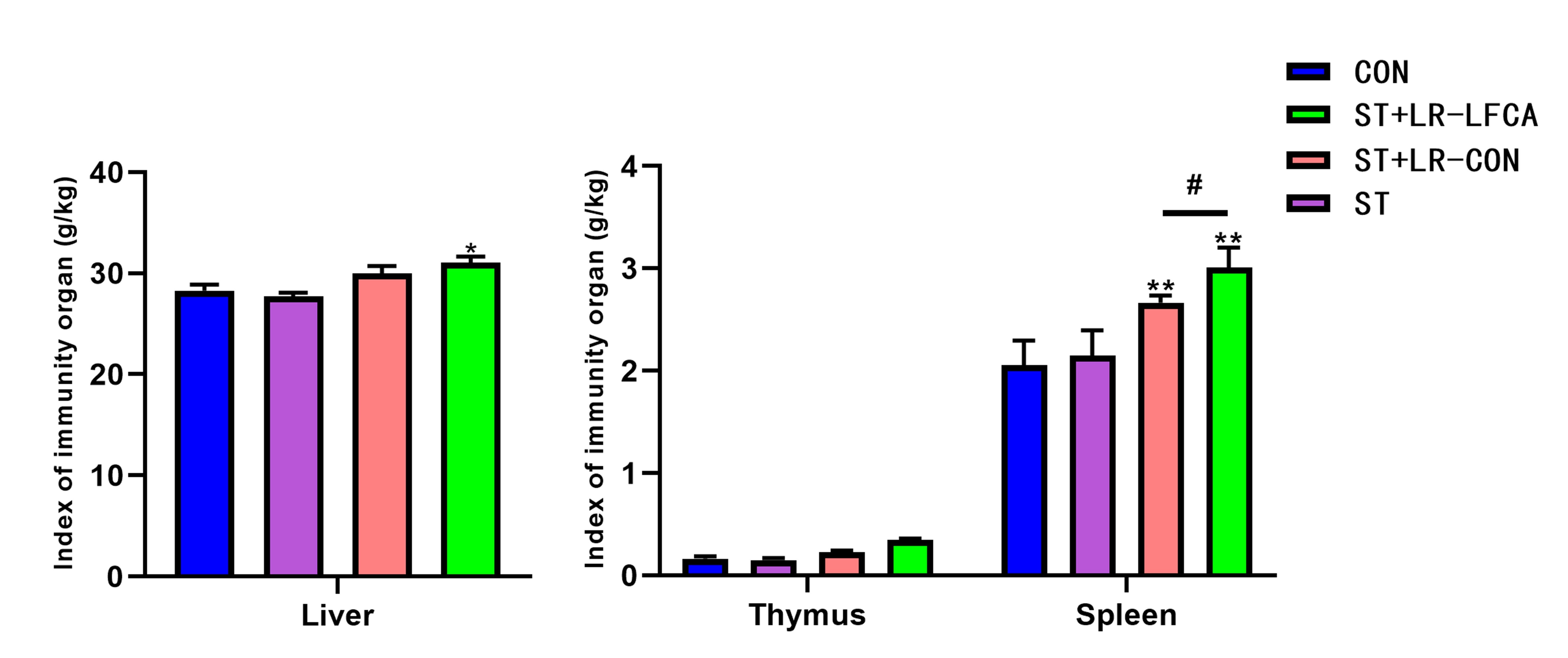


**Figure S3.** Impact of *S. typhimurium* infection on organ index of piglets post-administration with microencapsulated LR-LFCA. The relative weights of the organs (spleen, thymus, and liver) were calculated using the following formula: relative organ weight = organ weight (g) / terminal body weight (kg). Data are presented as the mean ± SD. *P< 0.05 vs ST; **P< 0.01 vs ST; #P< 0.05 vs ST+LR-LFCA.

**Figure S4.** Effect of administering microencapsulated LR-LFCA on the pathological changes in the tissues in *S. typhimurium*-infected piglets. Pathological analyses of the livers, spleens, and lung tissues as examined using H&E staining.


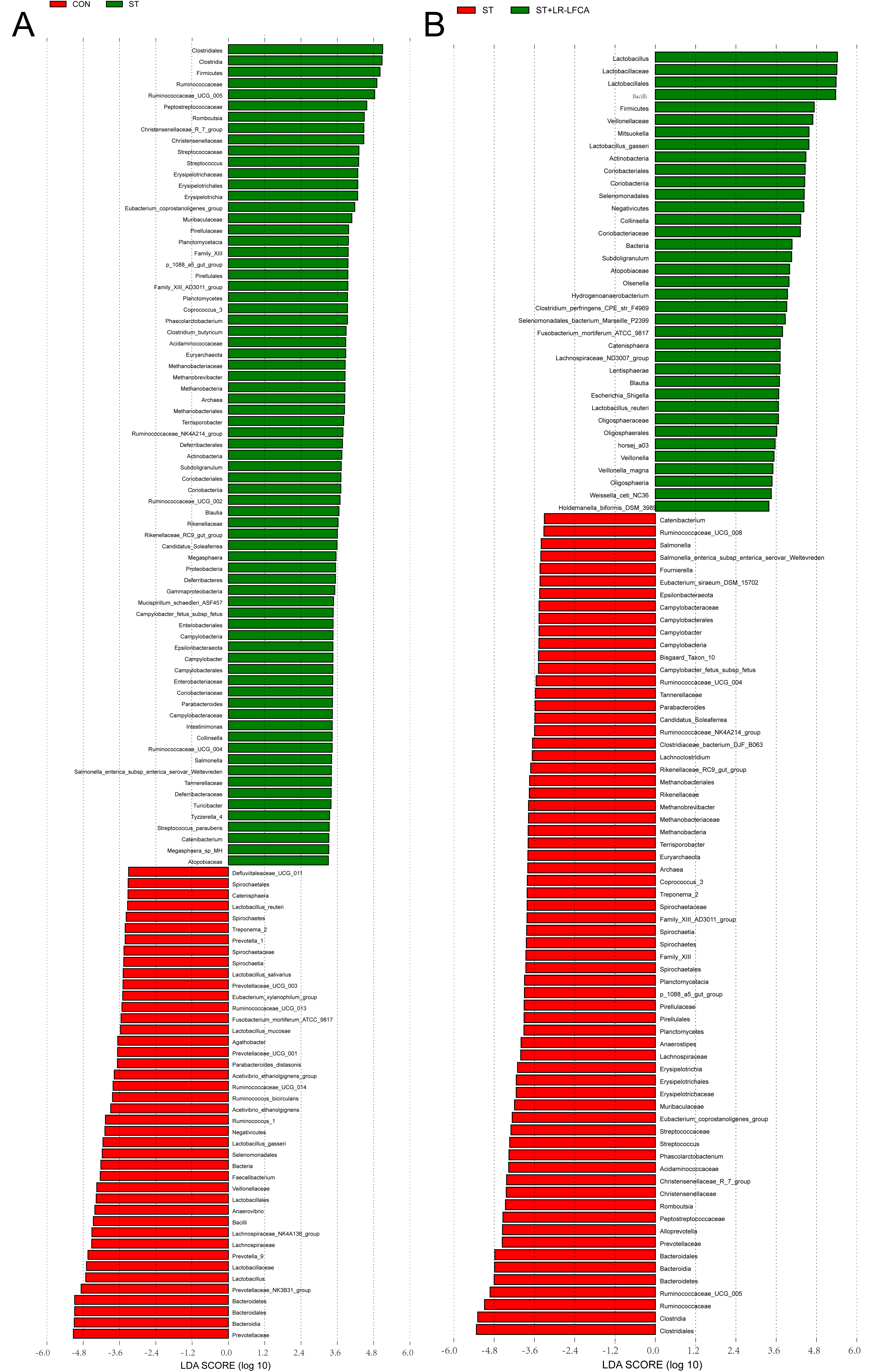


**Figure S5.** Changes in the intestinal microbial diversity in *S. typhimurium*-infected piglets after oral administration of microencapsulated LR-LFCA. (A) Cladogram plot of the LefSe analysis between the CON and ST groups. (B) Cladogram plot of the LEfSe analysis between the ST and ST+LR-LFCA groups.


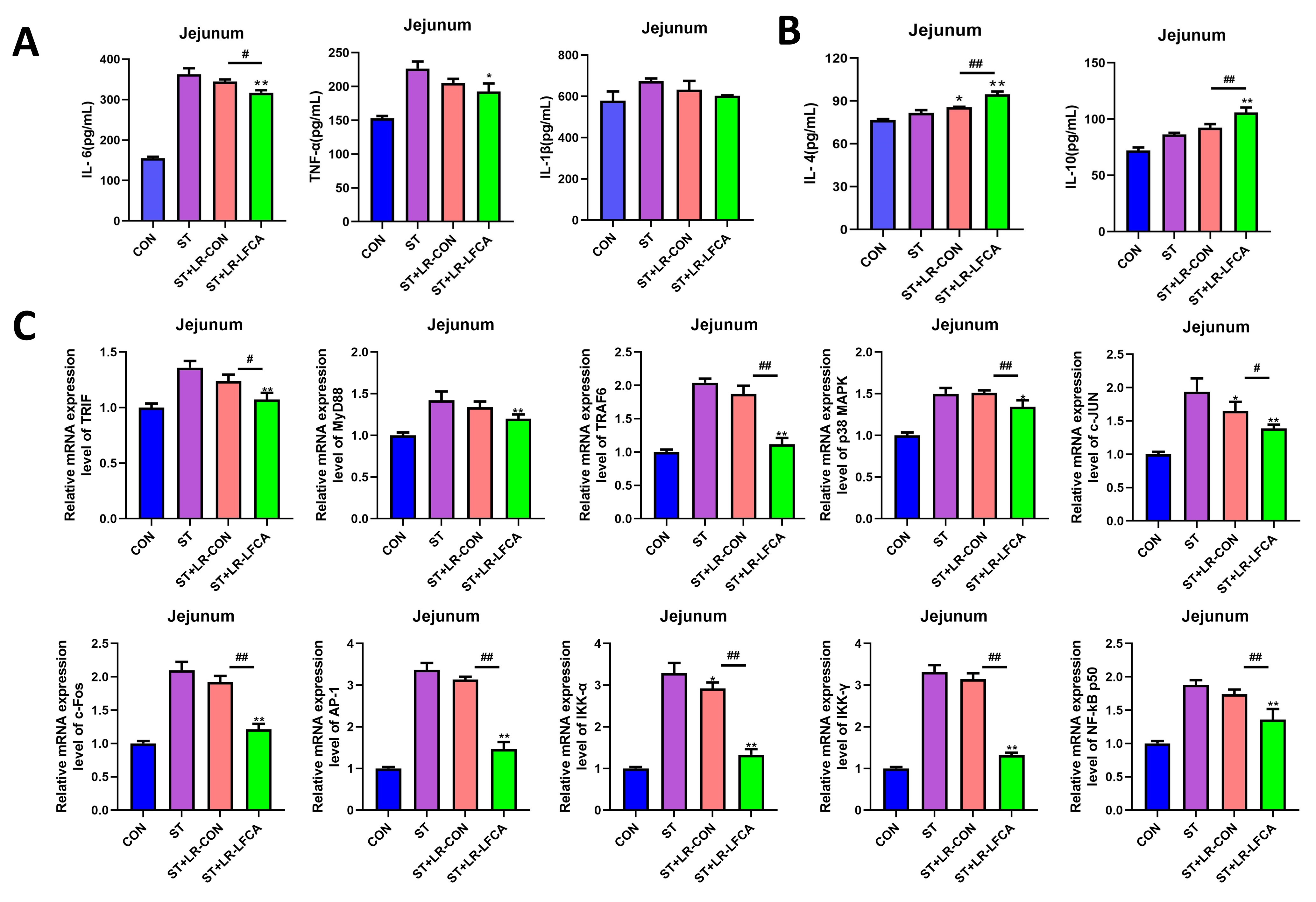


**Figure S6.** Impact of *S. typhimurium* infection on inflammatory factors in jejunum mucosa post-administration with microencapsulated LR-LFCA (A) IL-6, TNF-α, IL-1β, (B) IL-4, and IL-10 protein levels in the jejunum as measured in triplicate using ELISA. (C) TRIF, MyD88, TRAF6, p38 MAPK, c-JUN, c-Fos, AP-1, IKK-α, IKK-γ, and NF-κB p50 relative mRNA expression in the jejunum as measured in triplicate using real-time PCR. Data are presented as the mean ± SD. *P< 0.05 vs ST; **P< 0.01 vs ST; #P< 0.05 vs ST+LR-LFCA; ##P<0.01 vs ST+LR-LFCA.
